# 70329 Automated Lungs Segmentation and Airways Skeletonization from CT Scans in Patients with Cystic Fibrosis

**DOI:** 10.1017/cts.2021.521

**Published:** 2021-03-30

**Authors:** Juan A. Chong Chie, Paul R. Territo, Paul Salama

**Affiliations:** 1 Department of Electrical and Computer Engineering, Indiana University-Purdue University Indianapolis; 2 Department of Medicine, Division of Clinical Pharmacology, Indiana University School of Medicine

## Abstract

ABSTRACT IMPACT: Improve healthcare of patients with Cystic Fibrosis by reducing the time needed to generate results. OBJECTIVES/GOALS: We developed an automated framework capable of segmenting the lungs, extract the airways, and create a skeletonize map of the airways from CT scans of Cystic Fibrosis patients. As future expansion, the framework will be expanded to measure the airways diameters, detect the abnormal airways, and count the number of visible airways generations. METHODS/STUDY POPULATION: For this study, 35 CT scans from CF patients with different levels of severity were used to test the developed framework. The lungs segmentation was performed using an algorithm based on Gaussian Mixture Models for mild cases, and for severe cases a technique that uses convex hull and the recurrent addition of ‘dots’ was implemented. The airways extraction was performed using a 26-points connected components algorithm in conjunction with a curve fitting technique over the histogram of voxel values. Medial axis transform was used to perform the skeletonization of the extracted airways, and airways diameters determined via ray-casting. RESULTS/ANTICIPATED RESULTS: The framework was able to correctly obtain the segmented lungs in all 35 sample volumes regardless of disease severity. In contrast, it tends to fail to skeletonize the airways for severe cases where the framework is unable to differentiate between abnormal lungs conditions and dilated airways. Fine tuning is required to achieve better results. The expected result of the future implemented sections of the framework are focused to characterize the extracted airways by: 1) measuring the airways diameters; 2) detect and count the number of abnormal airways sizes; and 3) count the number of visible airways branching which will permit determination of stage and grade of the lungs of CF patients. DISCUSSION/SIGNIFICANCE OF FINDINGS: The proposed framework allows a fast and reproducible way to segment the lungs and create a skeletonized map of the airways that are independent of clinical training. In addition, this framework will be extended to obtain measurements of airway dilation and branching level, which could provide a deeper insight of the airways in CF patients.

